# Loss of zfp36 expression in colorectal cancer correlates to wnt/ β-catenin activity and enhances epithelial-to-mesenchymal transition through upregulation of zeb1, sox9 and macc1

**DOI:** 10.18632/oncotarget.10828

**Published:** 2016-07-24

**Authors:** Lucia Montorsi, Filippo Guizzetti, Claudia Alecci, Andrea Caporali, Andrea Martello, Claudio Giacinto Atene, Sandra Parenti, Silvia Pizzini, Paola Zanovello, Stefania Bortoluzzi, Sergio Ferrari, Alexis Grande, Tommaso Zanocco-Marani

**Affiliations:** ^1^ Department of Life Sciences, University of Modena and Reggio Emilia, Modena, Italy; ^2^ University/BHF Centre for Cardiovascular Science, University of Edinburgh, Edinburgh, UK; ^3^ Department of Molecular Medicine, University of Padova, Padova, Italy; ^4^ Department of Surgery, Oncology and Gastroenterology (DiSCOG), University of Padova, Padova, Italy; ^5^ Centre for Integrative Biology (CIBIO), University of Trento, Povo (Trento), Italy

**Keywords:** ZFP36, tristetraprolin, colon cancer, b-catenin, epithelial mesenchymal transition

## Abstract

The mRNA-destabilizing protein ZFP36 has been previously described as a tumor suppressor whose expression is lost during colorectal cancer development. In order to evaluate its role in this disease, we restored ZFP36 expression in different cell contexts, showing that the presence of this protein impairs the epithelial-to-mesenchymal transition (EMT) and induces a higher susceptibility to anoikis. Consistently, we found that ZFP36 inhibits the expression of three key transcription factors involved in EMT: ZEB1, MACC1 and SOX9. Finally, we observed for the first time that its expression negatively correlates with the activity of Wnt/β-catenin pathway, which is constitutively activated in colorectal cancer. This evidence provides a clue on the mechanism leading to the loss of ZFP36 in CRC.

## INTRODUCTION

Colorectal cancer (CRC) is a neoplasia affecting mucosa of large intestine and that develops through a multistage process resulting from the accumulation of multiple genetic mutations [1]. One of the most important alterations underlying CRC is the constitutive activation of Wnt/Δ-catenin signalling, which leads to nuclear translocation of Δ-catenin [2].

Epithelial-mesenchymal transition (EMT) is a process consisting in trans-differentiation of epithelial cells into motile mesenchymal cells. Even though it is a physiological process observed during development, wound healing and stem cell regulation, EMT is a hallmark of cancer progression as well [3]. Both in physiological and in pathological conditions, the transition follows a common and conserved programme, although having different characteristics depending on the tissue type. Independently from the context, EMT key events are the dissolution of epithelial cell-cell junctions and the gain of front-rear polarity in place of a basal-apical one. Furthermore, a reorganization of the cytoskeleton takes place, followed by changes in cell shape, acquisition of motility and, in many cases, the ability to degrade extracellular matrix [4]. All these events are supported by a down-regulation of epithelial-related genes’ expression in favour of an increased expression of mesenchymal specific genes. One of the most important hallmarks of EMT is the loss by cleavage of E-cadherin, a fundamental marker of epithelial phenotype, followed by the expression of N-cadherin, a known marker of mesenchymal phenotype. Wnt is one of the central pathways involved in promoting EMT in crosstalk with tyrosine kinase receptors, TGFΔ and Notch pathways. The gene expression changes contributing to EMT involve master regulators including SNAIL, TWIST and ZEB that are activated from the initial stages of transition [5–7]. Snail proteins (1 and 2) and ZEB transcription factors repress epithelial genes or induce mesenchymal gene expression by binding to E-box sequences through their DNA binding domains. TWIST proteins are basic helix-loop-helix (bHLH) transcription factors and, similarly to SNAIL, they down-regulate epithelial genes and activate mesenchymal genes’ expression. Other genes, such as Sex-determining Region Y box 9 (SOX9) and Metastasis-associated in colon cancer-1 (MACC1) are dysregulated in colon carcinoma and play a fundamental role at the onset of EMT [8, 9].

ZFP36 is the prototypic member of the TIS11/ZFP36 family of RNA destabilizing proteins [10]. The RNA-binding specificity resides in the central tandem zinc finger domain which interacts at a nanomolar affinity level with mRNA molecules containing in their 3′ untranslated region (3′UTR) ARE sequences mostly found in the form of the pentamer AUUUA or the nonamer UUAUUUAUU [10]. The discovery of ZFP36′s role as an mRNA decay factor firstly came from the characterization of ZFP36 specific knockout mice, which showed a chronic inflammatory phenotype. Further evidences followed showing the ability of ZFP36 to destabilize mRNAs encoding for several pro-inflammatory cytokines [11]. More recently, ZFP36 has been linked to cancer thanks to evidences showing its down-regulated state in several tumours including CRC, and the demonstration of its ability to regulate the expression of many oncogenic targets carrying ARE sequences in their 3′ UTR [12, 13]. In other words, although there is no evidence showing that the gene encoding for ZFP36 is a target of genomic loss or rearrangement, it appears to behave as a tumour suppressor. Particularly, it has been very recently demonstrated that ZFP36 is capable of inducing a shift from mesenchymal to epithelial phenotype in different cancer cell lines through the down-regulation of TWIST1 and SNAIL1 [14].

In this study, we investigated the biological meaning of the loss of ZFP36 in CRC. Specifically, we confirmed that by restoring ZFP36 expression in different CRC cell lines, EMT appears to be inhibited while a higher susceptibility to anoikis occurs. This evidence depends on the downregulation of different ZFP36′s oncogenic targets among whom we validated SOX9, MACC1 and ZEB1 that were never described previously as ZFP36 targets. Moreover, in the absence of genomic loss, we provide evidence suggesting that ZFP36 downregulation in CRC is inversely correlated to Wnt/β-catenin constitutive activation, providing new insights into loss of post-transcriptional regulatory circuits during CRC tumour development and progression.

## RESULTS

### ZFP36 expression in colon cancer

**Figure 1:**
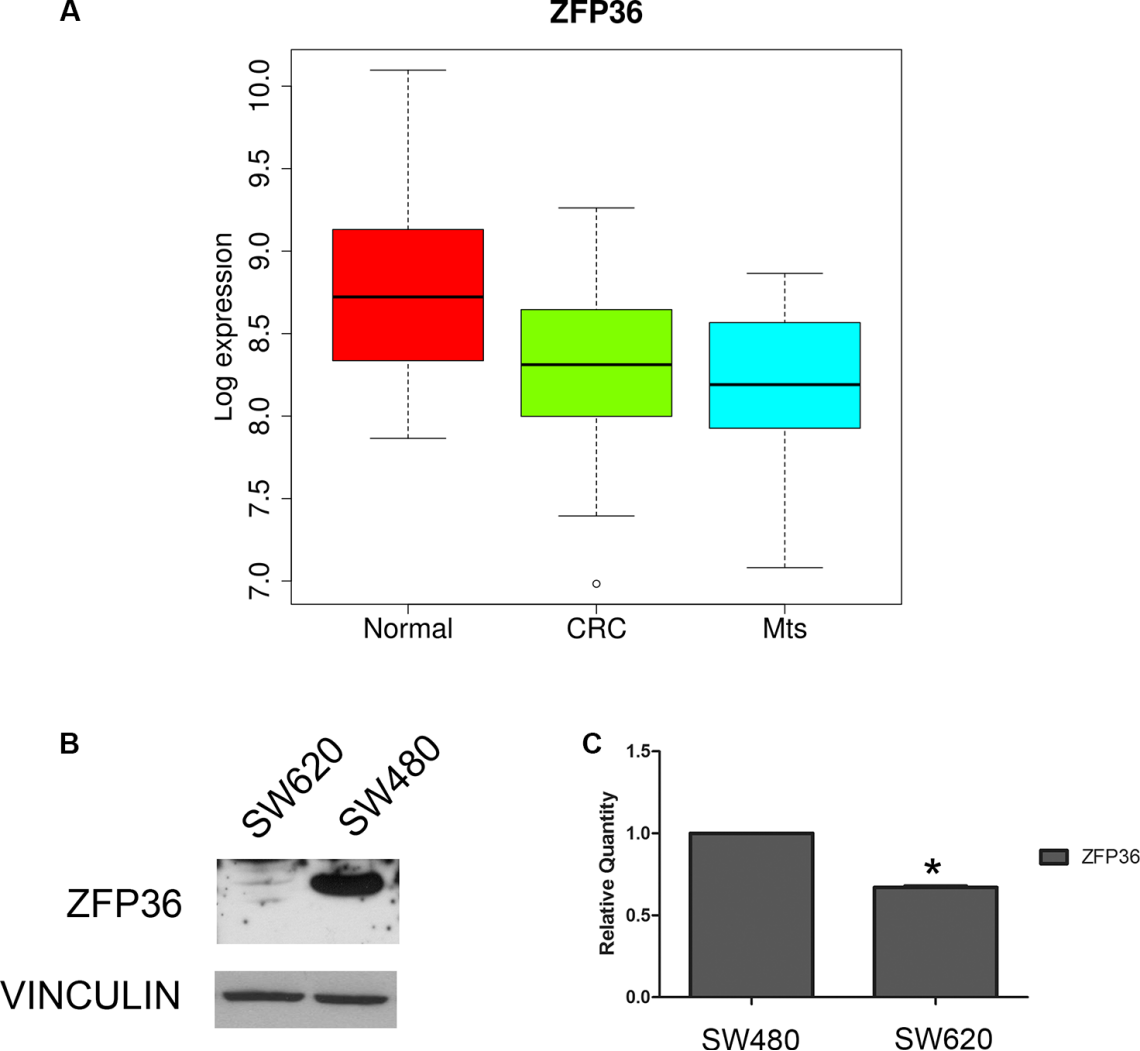
Analysis of ZFP36 expression in CRC-related samples (Panel **A**) Boxplot of ZFP36 mRNA Log2 expression levels evaluated in a GEO database of gene expression profiles derived from 23 normal colon mucosa (Normal), 30 primary colon carcinoma (CRC) and 27 liver metastases (Mts) samples. The thick line indicates the median value, the coloured box indicates the interquartile range and the whiskers the minimum and maximum values excluded outliers. Open circles represent data points outside the whiskers. (Panel **B**) ZFP36 protein expression in CRC cell lines derived from primary tumour (SW480) and matched metastatic lesion (SW620). Vinculin was used as loading control. (Panel **C**) ZFP36 mRNA levels detected in SW480 and SW620 cell lines through qRT-PCR. Results are represented as means of two experiments (+/− SEM) and GAPDH was used as endogenous control. *p < 0.05.

We analysed ZFP36 expression in a specific dataset described in Materials and Methods. According to gene expression profiles, in the 80 samples considered – composed of 23 normal colon mucosa (Normal), 30 primary colon carcinoma (CRC) and 27 liver metastases (Mts) – the expression of ZFP36 gene is significantly decreased when comparing primary tumour with normal counterpart (Figure 1A). The same gene is further downregulated in metastases compared to both primary tumour and the normal counterpart (Supplementary Table S1). The median log2 expression value of ZFP36 decreases from 8.7 of normal to 8.3 of CRC and to 8.2 of liver metastasis. As shown in table Supplementary Table S2, ZFP36 results significantly differentially expressed in CRC vs Normal and Mts vs Normal comparisons from differential expression tests. However, it does not result significantly differentially expressed in Mts vs CRC comparison, even though the tendency toward decrease is constant when considering the progression represented by Normal, CRC and Mts samples. Figure 1B and 1C are respectively the results of an immunoblot and a real-time PCR; they both show that ZFP36 is expressed in the cell line SW480, which is derived from primary colon carcinoma, while it is strongly down-regulated in SW620, a cell line derived from a metastatic form of the same tumour. Altogether these results suggest that ZFP36 seems to undergo a more pronounced down-regulation in metastases rather than in primary tumours compared to normal tissues.

### ZFP36 ectopic expression determines a shift from mesenchymal to epithelial phenotype

**Figure 2:**
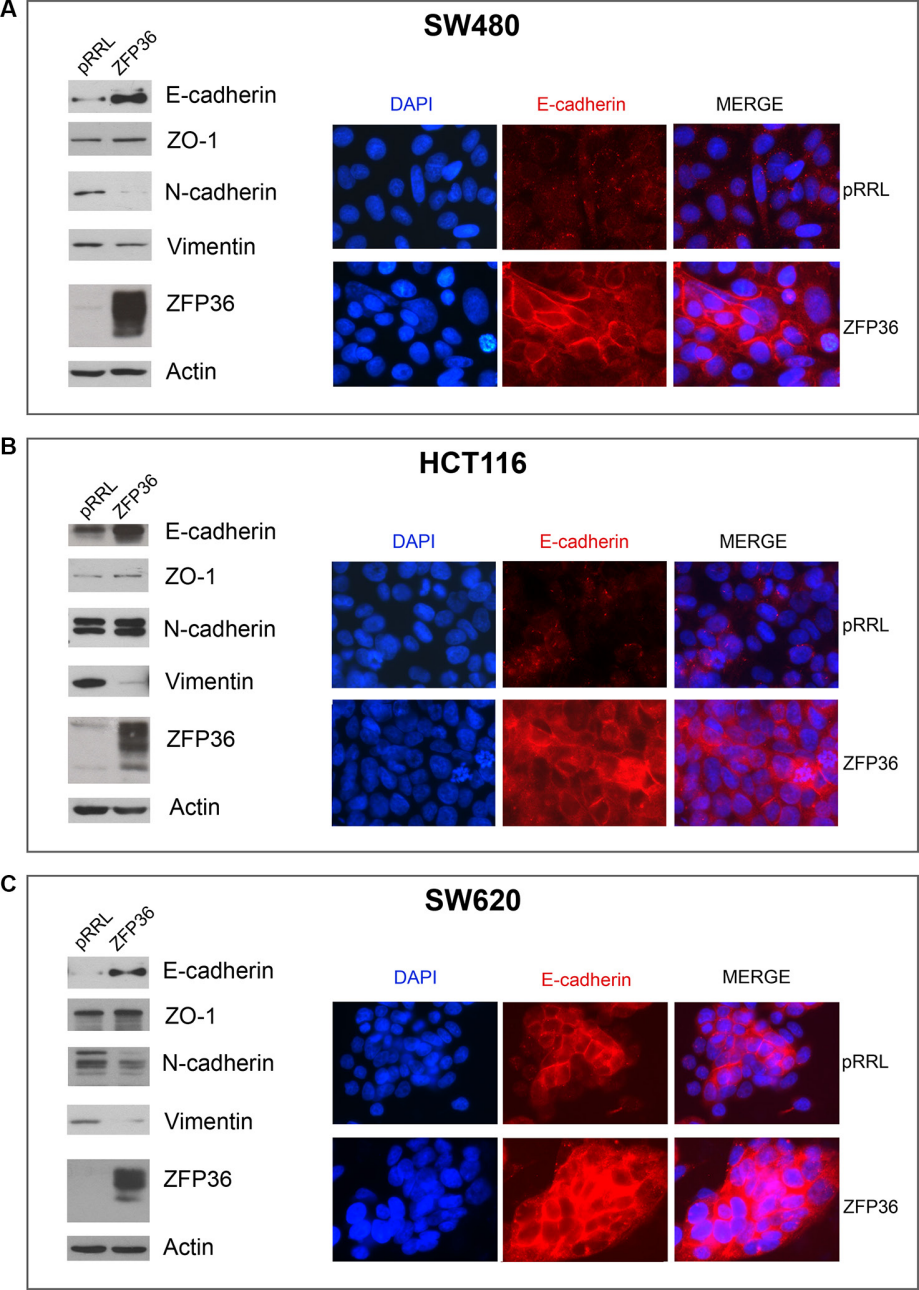
Analysis of epithelial markers in ZFP36 overexpressing cells SW480 (Panel **A**), HCT116 (Panel **B**) and SW620 (Panel **C**) were infected with empty vector (pRRL) or ZFP36-overexpressing vector (ZFP36) and the corresponding total protein lysates were analysed through Western blot using antibodies against E-cadherin, ZO-1, N-cadherin, Vimentin and ZFP36. Actin was used as a loading control. The same cells were seeded on glass dishes and subsequently subjected to immunofluorescence analysis with antibody against E-cadherin (red) to assess its sub-cellular localization. Nuclei were counterstained with DAPI (blue).

Figure 2 shows Western blot analysis of EMT markers E-cadherin, Zona Occludens 1 (ZO-1), N-cadherin and Vimentin in three CRC cell lines (SW480, SW620 and HCT116) following ZFP36 ectopic expression. It appears that ZFP36′s increase of expression determines a down-regulation of mesenchymal markers N-cadherin and Vimentin and the up-regulation of epithelial markers E-cadherin and ZO-1 (densitometric analysis reported in Supplementary Figure S1). Figure 2, panels A, B and C also display an immunofluorescence showing E-cadherin expression following ZFP36 restoration in SW480, HCT116 and SW620 cell lines. Images show that, following ZFP36 over-expression, E-cadherin results to be increased and clearly localised on the cell membrane.

**Figure 3:**
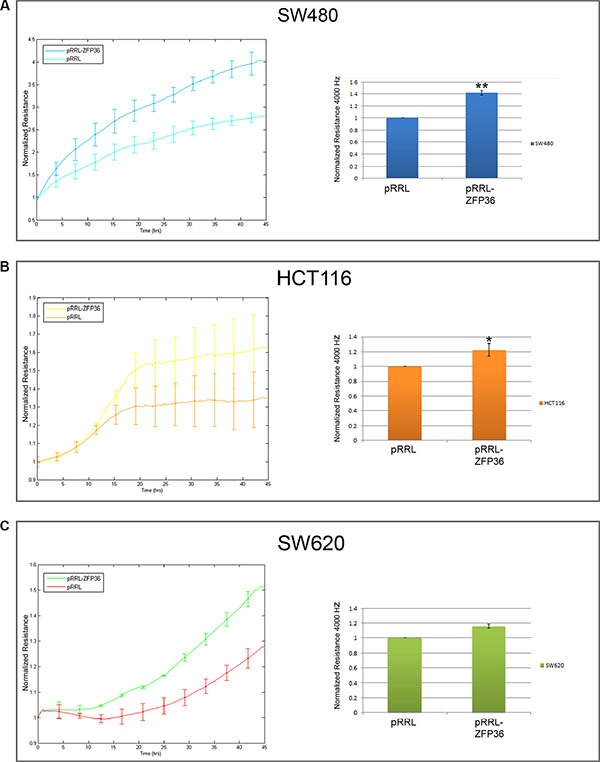
ZFP36 overexpression increases epithelial barrier function SW480 (Panel **A**), HCT116 (Panel **B**) and SW620 (Panel **C**) infected with empty vector (pRRL) or ZFP36-overexpressing vector (pRRL-ZFP36) were seeded on uncoated gold microelectrodes and subjected to a small alternating current. Immediately after seeding, resistance at 4000 Hz was recorded every 5 minutes for at least 45 hours. Graphs on the left-hand side of the panel show the mean of the normalized resistance for three different experiments plotted in function of time (+/−SEM). The histograms on the right-hand side of each panel summarize the results described in the graphs: approximately 48 hours after seeding, resistance at 4000 z became stable and was recorded for further 5 hours. Values obtained during the last 5 hours were averaged and normalized on pRRL values. *p < 0.05, **p < 0.001.

To test whether the activity of intercellular junctions was increased by ZFP36, we performed impedance-based experiments aimed to measure the barrier function of the cellular monolayer. Thus, we recorded the resistance opposed by the cellular monolayer to an electric field of 4000 Hz, which represents the best condition in order to assess the strength of intercellular epithelial junctions in this kind of experiments. In this setting, electric current preferentially flows in the space between cells, which is usually closed by intercellular contacts in the case of an epithelial monolayer. Therefore, the stronger the cellular junctions, the higher the resistance contrasting the flow. The results of these experiments performed on SW480, HCT116 and SW620 cells are shown in Figure 3, panels A, B and C. They suggest that ZFP36-overexpressing cells exhibit an augmented resistance of the cellular monolayer in comparison to control cells. Changes in the normalised resistance in function of time are reported in the graphics on the left-hand side of panels A–C, where 0 h corresponds to the time after seeding. In the graphics on the right-hand side of the same panels, the same parameter is recorded for 5 hours after the samples had reached the complete cellular confluence, and then averaged and expressed as a fold change. Precisely, cellular resistance significantly increases of the 40% in SW480, of 20% in HCT116 and of 20% in SW620 cells.

### ZFP36 ectopic expression inhibits colony formation, growth in suspension and migration capability of CRC cell lines

**Figure 4:**
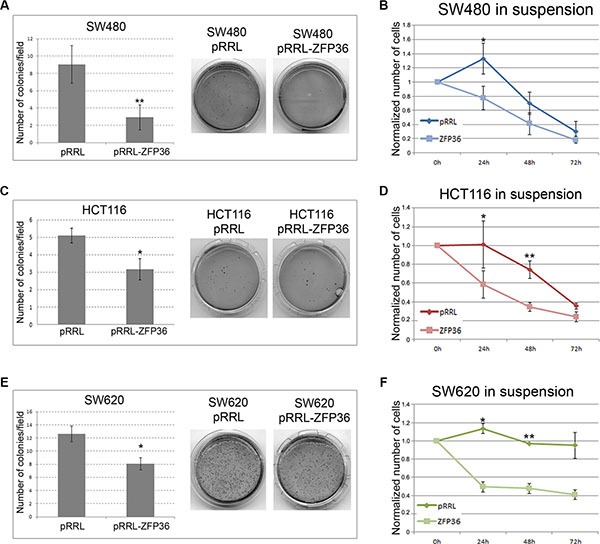
ZFP36 overexpression impairs anchorage-independent-growth and sensitizes to anoikis SW480 (Panel **A**), HCT116 (Panel **C**) and SW620 (Panel **E**) infected with empty vector (pRRL) or ZFP36-overexpressing vector (pRRL-ZFP36) were seeded in soft-agar medium (DMEM + 0.3% Agar) and allowed to form colonies. The efficiency in colonies formation was then evaluated by randomly selecting 10 optical fields for each plate and calculating the average number of colonies. The histograms represent these figures as the mean of three independent experiments +/−SEM. Photographs show the crystal-violet staining of one representative experiment. In (panels **B**, **D** and **F)** equal amounts of respectively SW480, HCT116 and SW620 infected with pRRL or ZFP36 vectors were seeded in presence of Dispase to prevent cell adhesion (in suspension). Vital cells were then counted through Trypan blue exclusion assay. Results were normalized on the number of seeded cells and reported as the mean of three independent experiments (+/− SEM). *p < 0.05, **p < 0.001.

Figure 4, panels A, C and E shows the results of several soft agar assays performed on SW480, HCT116 and SW620 cell lines showing that ZFP36 over-expression determines a drastic decrease in colony formation. Figure 4, panels B, D and F shows the results of a Dispase assay suggesting that CRC cell lines ectopically expressing ZFP36 have a lower capability of growing in the absence of a substrate compared to controls, therefore displaying a higher susceptibility to anoikis.

**Figure 5:**
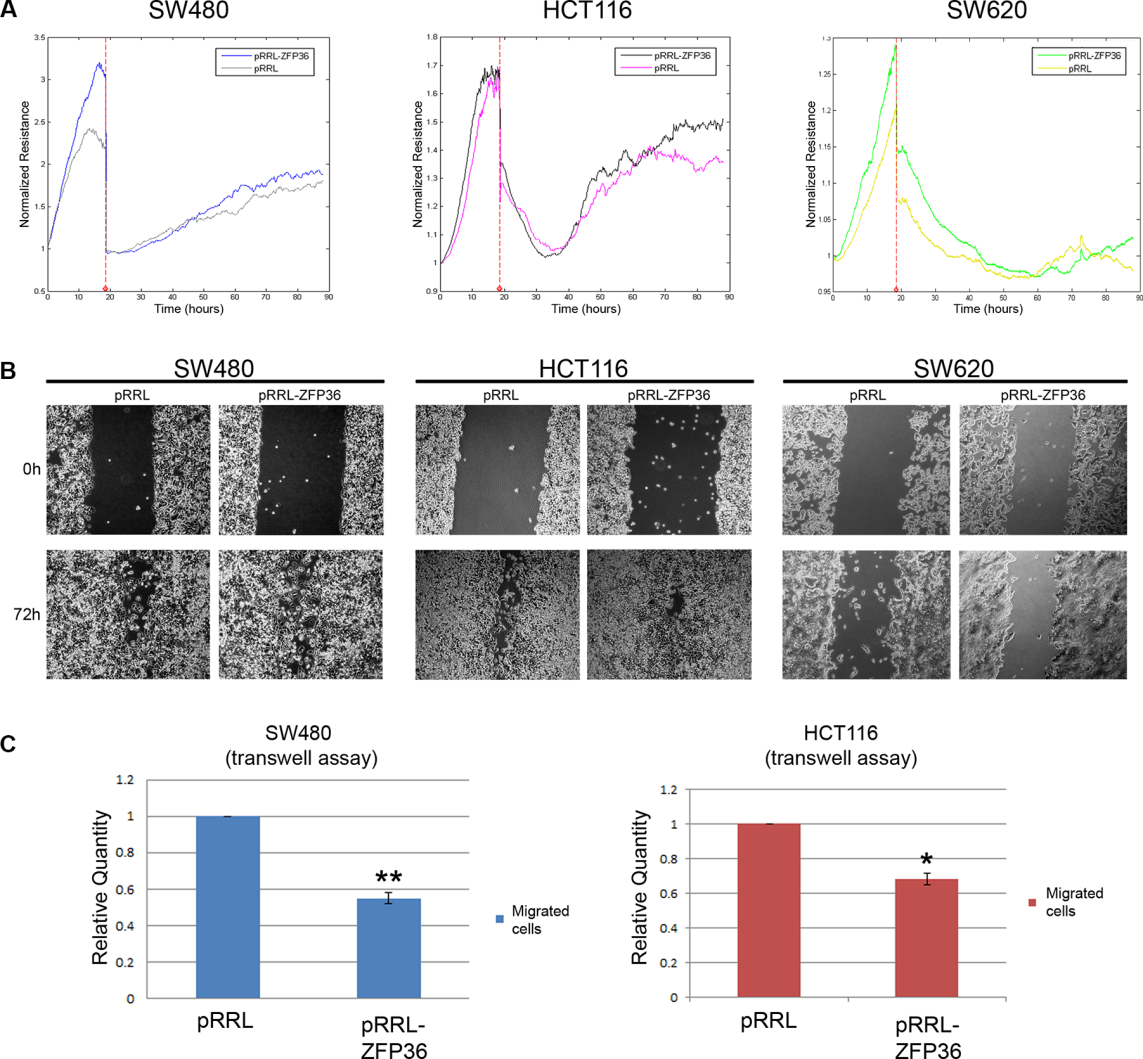
ZFP36 restoration in CRC cell lines exerts minor effects on cellular migration (Panel **A**) SW480, HCT116 and SW620 infected with empty vector (pRRL) or ZFP36-overexpressing vector (pRRL-ZFP36) were seeded on uncoated gold microelectrodes and subjected to a small alternating current. After reaching complete confluence, a wound in the cellular layer was produced by applying a high voltage pulse and the recovery of the cellular monolayer was monitored by measuring Resistance at 4000 Hz. The graphs shown represent a single experiment. (Panel **B**) SW480, HCT116 and SW620 infected with empty vector (pRRL) or ZFP36-overexpressing vector (pRRL-ZFP36) were grown until complete confluence, and a scratch on cellular monolayer was performed with a pipette tip. The results of one representative experiment are shown in the photographs. (Panel **C**) SW480 and HCT116 infected with empty vector (pRRL) or ZFP36 overexpressing vector (pRRL-ZFP36) were serum-starved and seeded inside the upper chamber of 8 μm-pore transwell insert. Cells were then allowed to migrate towards a chemo-attractant stimulus (FBS) for 16 hours. Migrated nuclei were stained with DAPI and quantified by taking random pictures. Results obtained were normalized on the pRRL values and represented as the mean of three independent experiments.

Another important feature of cells undergoing EMT is the acquisition of migratory abilities. For this reason, we speculated that ZFP36 overexpression could decrease cellular motility. Surprisingly, the results of an assay performed with the ECIS machinery (Figure 5A) – where the wound in cellular monolayer was achieved by a high voltage pulse, and the efficiency of gap closure was assessed by measuring the cellular impedance – demonstrated that, comparing control cells and ZFP36 overexpressing cells, there is no significant difference in the time required for the gap to close. Even a wound healing assay, shown in Figure 5B, did not provide clear evidence that SW480 or HCT116 over-expressing cells are slower in repairing the wound compared to their controls. To further investigate this matter, we performed a transwell assay. Results (Figure 5C) show a difference in the migration capability of control cells and ZFP36 expressing cells. Indeed, the count of migrated nuclei shown in panel C, reveals that both SW480-ZFP36′s and HCT116-ZFP36′s migration efficiency is halved compared to control cells. The same assay on SW620 cells is not shown since these cells do not have migration ability on transwell. Altogether, these data suggest that ZFP36 expression has higher impact on anoikis and anchorage independent growth rather than on migration.

### MACC1, ZEB1 and SOX9 are directly targeted by ZFP36

**Figure 6:**
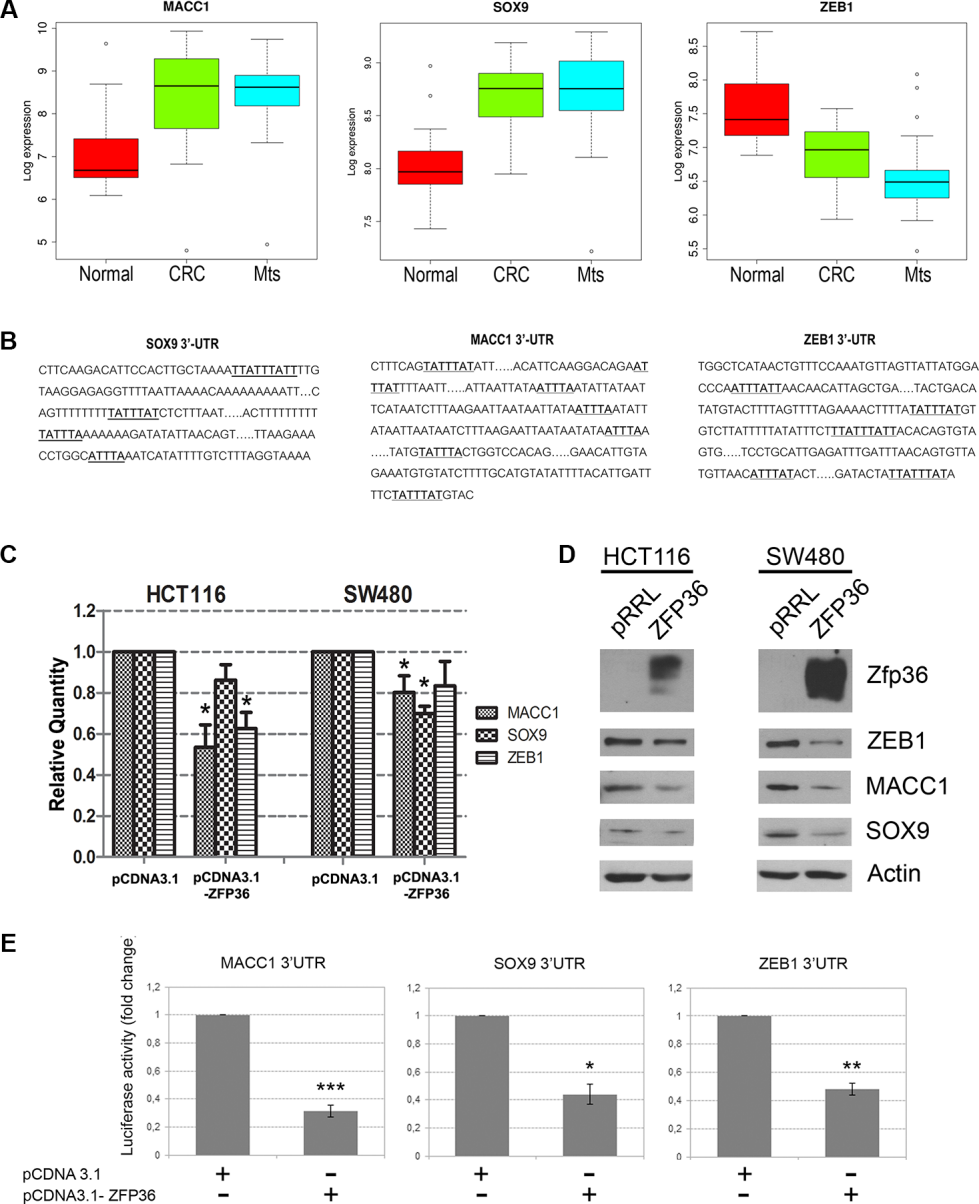
Validation of three novel ZFP36 target genes involved in EMT (Panel **A**) Boxplot of Log 2 expression values of MACC1, SOX9 and ZEB1 in 23 normal colon mucosa (Normal), 30 primary colon carcinoma (CRC) and 27 liver metastases (Mts) samples. The thick line indicates the median value, the coloured box indicates the interquartile range and the whiskers the minimum and maximum values excluded outliers. Open circles represent data points outside the whiskers. (Panel **B**) Schematic representation of the 3′UTRs sequences of MACC1, SOX9 and ZEB1. A-U rich sequences (ARE) are highlighted in bold. (Panel **C)** HCT116 and SW480 cells were transfected with an empty vector (pCDNA3.1) or a ZFP36-overexpressing vector (pCDNA3.1-ZFP36). RNA was extracted after 48 hours and MACC1, SOX9, ZEB1 mRNA levels were analysed through qRT-PCR analysis. Results are represented as means of three experiments (+/−SEM) and GAPDH was used as endogenous control. *p < 0.05. (Panel **D**) HCT116 and SW480 were infected with an empty vector (pRRL) or a ZFP36-overexpressing vector (ZFP36) and corresponding total protein lysates were analysed through Western blotting techniques with antibodies against ZEB1, MACC1, SOX9 and ZFP36. Actin was used as loading control. (Panel **E**) A fragment of the 3′UTRs of MACC1, SOX9 and ZEB1 was cloned in a pGL3 vector, downstream of the Luciferase gene. These constructs where co-transfected with a Δ-gal reporter plasmid and with an empty vector (pCDNA3.1) or ZFP36 overexpressing vector (pCDNA3.1-ZFP36) in HEK293T cells. Cells were harvested after 48 hours, luciferase activity was measured and normalized over Δ-gal signals. Results are represented as means of three independent experiments +/−SEM. *p < 0.05, **p < 0.001, ***p < 0.0001.

Figure 6, panel A shows the expression of MACC1, ZEB1 and SOX9 in the CRC dataset previously described. The expression levels of such genes were analysed since they all carry an ARE sequence in the 3′UTR region of their mRNAs (Figure 6, panel B) and therefore can be considered as putative ZFP36 targets. MACC1 and SOX9 show a progressive increase in tumour and metastases compared to normal tissue with a median log 2 expression value increase from 6.8 to 8.7 in normal cells, and from 8.0 to 8.8 in primary tumour cells (Supplementary Table S2). On the contrary, ZEB1 expression seems to decrease in tumour versus normal (median log2 expression value from 7.6 of normal to 6.9 of tumour and 6.6 of metastasis). The latter observation is controversial since ZEB1 is widely recognized in literature as upregulated in CRC and to be responsible for EMT [15–18].

Figure 6, panel C is a real time PCR analysis showing that expression of ZEB1, MACC1 and SOX9 mRNA decreases in HCT116 and SW480 cells following ZFP36 over-expression. Panel D shows Western blots confirming at the protein level that the expression of the putative targets decreases in HCT116 and SW480 cells following ZFP36 ectopic expression (densitometric analysis reported in Supplementary Figure S2).

To demonstrate that MACC1, ZEB1 and SOX9 are directly targeted by ZFP36, we performed a luciferase reporter assay, generating luciferase reporter constructs (pGL3-based) allowing transcription of a luciferase mRNA carrying the 3′ UTRs of MACC1, ZEB1 and SOX9. The results are described in Figure 6, panel E; they show that co-expression of the three vectors encoding the different 3′UTRs with a vector expressing ZFP36 impairs protein production by either promoting mRNA degradation or by inhibiting mRNA translation, thus suggesting that ZFP36 is capable of directly binding and therefore destabilizing the mRNAs of MACC1, ZEB1 and SOX9.

### ZFP36 expression in CRC cell lines is inversely correlated to Wnt/*β*−catenin pathway activation

**Figure 7:**
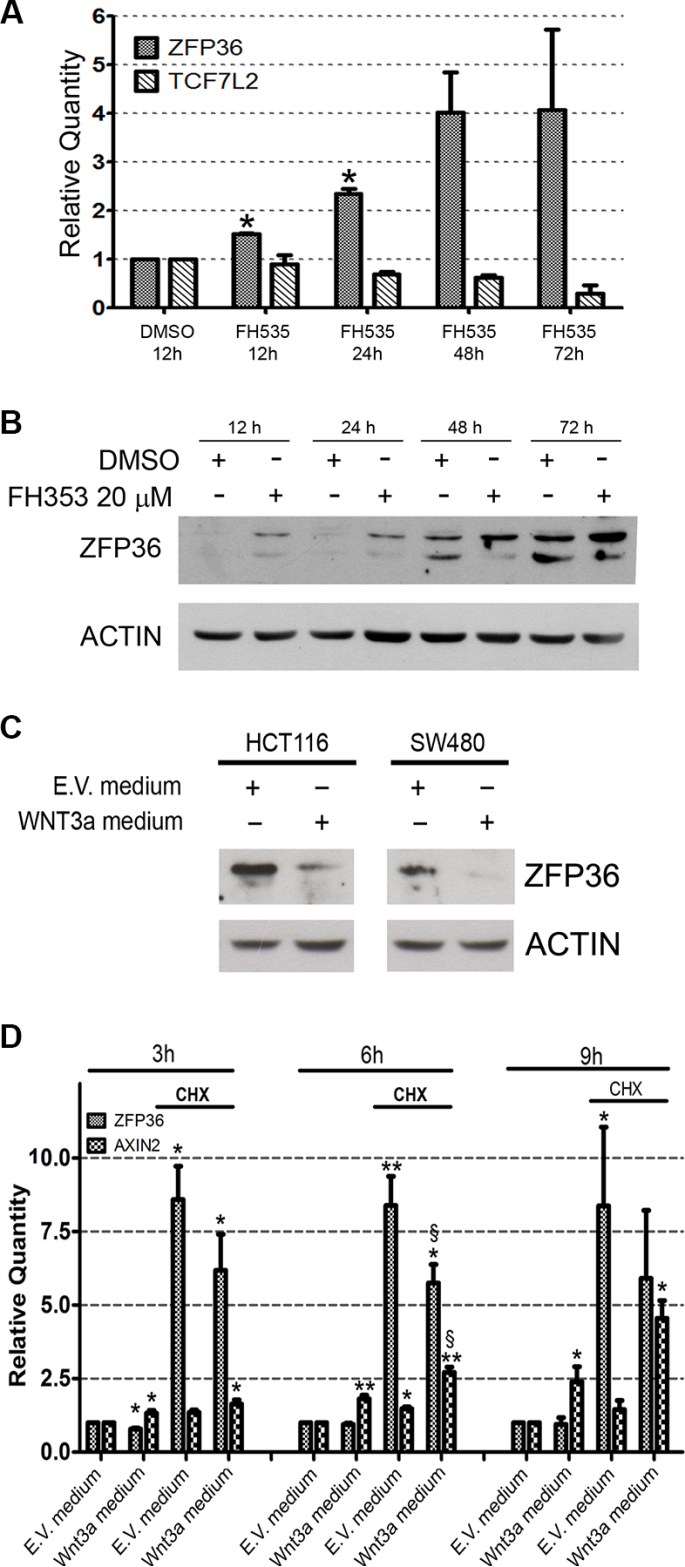
Wnt/β-catenin pathway inversely regulates ZFP36 expression (Panel **A**) HCT116 were treated with FH353 20 μM or vehicle (DMSO) for the indicated time. RNA was extracted, retro-transcribed and used for real-time qRT-PCR analysis of ZFP36 and TCF7L2 transcripts. Results were normalized on DMSO values at each time point. Histograms represent the mean of two independent experiments (+/−SEM), GAPDH was used as endogenous control. *p < 0.05. (Panel **B**) HCT116 treated as in panel A were lysed and equal amounts of total protein extracts were loaded on a SDS-PAGE and subsequently analysed through Western blot techniques with an antibody against ZFP36. Actin was used as loading control. (Panel **C**) HCT116 and SW480 were treated with conditioned medium obtained from empty vector cells (E.V medium) or Wnt3a overexpressing cells (Wnt3a medium). After 24 hours, cells were collected, lysed and equal amounts of protein were loaded on SDS-PAGE and subsequently analysed through Western blot technique with an antibody against ZFP36. Actin was used as loading control. (Panel **D**) HCT116 were treated with E.V. medium and Wnt3a medium supplemented with Cyclohexymide (CHX) or DMSO (not indicated). After the indicated amount of time, RNA was extracted, retro-transcribed and used for real-time PCR analysis of ZFP36 and Axin2 transcripts. Results were normalized on E.V. medium values at each time point. Histograms represent the mean of four independent experiments (+/−SEM), GAPDH was used as endogenous control. *= *p* < 0.05, **= *p* < 0.001. §= *p* < 0.05 if *t*-test was performed on the values obtained from Wnt3a-CHX treated cells in comparison to E.V medium-CHX treated cells.

In the absence of genomic loss or described mutations, we investigated whether the down-regulation of ZFP36 observed in CRC might depend on the activity of specific pathways. Since Tcf/β-catenin abnormal signalling is a hallmark of CRC, we looked for a relation between this pathway and ZFP36 expression. To do so, we used the Tcf/β-catenin signalling inhibitor FH535. Figure 7, panel A displays a real time PCR showing that administration of FH535 determines an increase in ZFP36 expression. Panel B is a Western blot showing that FH535 administration increases ZFP36 expression also at the protein level.

In order to further clarify the relation between Tcf/β-catenin signalling and ZFP36 expression, HCT116 cells were cultured with a medium enriched in Wnt3a concentration. Although this cell line expresses very low levels of ZFP36, administration of Wnt3a determines a further decrease of its expression together with an increase of Axin2 expression, which is a well-known target of β-catenin signalling (Figure 7C).

These observations generate two main hypotheses: the first is that ZFP36 transcription could be downregulated by a repressor induced by Wnt3a treatment, while the second one is that β-catenin could directly repress ZFP36. In the attempt to clarify this issue, we took advantage of Cycloheximide (CHX), a molecule that inhibits protein synthesis. Evaluation of expression levels of Axin2, a well-known β-catenin target gene, was used as positive control of the effectiveness of Wnt3a usage. Treatment with Wnt3a conditioned medium alone for 3, 6 and 9 hours resulted in a time dependent increase of Axin2 transcripts and to a slight decrease in ZFP36 mRNA (Figure 7D). The addition of CHX in culture medium surprisingly resulted in a strong increase of ZFP36 mRNA (Figure 7D), a phenomenon previously described in two other papers and not linked to an increase in protein levels [19, 20]. Interestingly, also in presence of CHX, Wnt3a-treated cells displayed reduced levels of ZFP36 transcript compared to cells receiving E.V. medium, with a decrease of 30% after 6 hours of treatment (Figure 7D). These data demonstrate that inhibition of protein synthesis does not affect Wnt3a-mediated repression of ZFP36, thus suggesting that this could be a direct effect of β-catenin/TCF complex. Another clue in favour of this hypothesis is represented by the presence of some TCF7L2 putative binding sites in the promoter of ZFP36, and by the proof of the effective binding of TCF7L2 to some of these sequences, provided by Encode project data (https://genome.ucsc.edu/ENCODE/).

## DISCUSSION

ZFP36 was originally described as an active regulator of the inflammatory response. Today several publications show that its expression is commonly deficient in a large number of cancer cell types when compared to normal cells [21]. Moreover, its expression seems to counteract malignant progression by interfering with different pathways, depending on the disease model [22–24].

In this work we described three new ZFP36 targets, each one containing ARE sequences in their 3′ UTR: ZEB1, SOX9 and MACC1. These genes are well known for being deregulated in CRC and for being responsible of epithelial to mesenchymal transition in synergy with Twist1 and Snail1 [5–9], genes which were very recently described as ZFP36 targets as well [14].

From a functional point of view, we confirmed that, by negatively regulating its targets’ expression, ZFP36 restoration has an impact on EMT and, as described in the results section with reference to the ECIS and immunoflurescence assays, it seems to be capable of bringing back the cells to a more epithelial-like phenotype. Indeed, these cells lose the ability to grow in soft agar and display a higher susceptibility to anoikis while, in our study, we observed a minor effect on the ability of ZFP36 to impinge on migration capacity.

Although it is quite recognized that ZFP36 expression is lost in several malignancies, much less is known concerning the mechanisms determining this event. Neither specific mutations have been described, nor genomic loss has been singled out. Epigenetic causes have been suggested [25]. However, it can be also hypothesised that, since loss of ZFP36 strongly fosters tumour progression, this event can occur in different cell contexts through the modulation of the pathways that are crucial to a specific tumour. To this regard, we observed that ZFP36 expression in CRC cell lines is inversely correlated to Wnt/β-catenin pathway activity. It remains to fully understand whether this depends directly on β-catenin or indirectly on the activity of another repressor activated by the pathway. Based on the CHX experiment shown in Figure 7, we are inclined towards the first hypothesis. Interestingly, there are only a couple reports describing the ability of β-catenin to act as a transcriptional repressor rather than as an activator [26–28]. The inverse trend between Wnt/β-catenin and ZFP36 is surely noteworthy and deserves wider studies that would help clarifying the mechanisms underlying the loss of ZFP36 in specific cancers.

## MATERIALS AND METHODS

### Expression data

We considered the expression profiles of ZFP36, MACC1, SOX9 and ZEB1 genes in colon cancer patients, using a dataset available at the GEO database (GSE35834). This dataset reports expression estimations, obtained with GeneChip Human Exon 1.0 ST (Affymetrix) platform, of 22517 genes in 80 samples, including 23 normal colon mucosa (Normal), 30 primary colorectal tumours (CRC) and 27 liver metastases (Mts) obtained from 46 patients diagnosed with colorectal carcinoma at TNM stage IV, who underwent surgery at the University of Padova (Surgery Unit, Department of Surgery, Oncology and Gastroenterology) in the period 1994–2008 [29, 30].

Differential expression analysis was performed using limma package, available from BioConductor for the R statistics environment (BioConductor, www.bioconductor.org), setting *p*-value < 0.05 and using Benjamini Hochberg (BH) method to adjust *p*-value.

### Cell culture and treatment

SW480 and SW620 cell lines were kindly gifted by Prof Alberto Bardelli, (University of Turin). HCT116 and HEK293T cells were purchased from ATCC. All cell lines were grown in DMEM supplemented with 10% FBS (Sigma), 2 mM L-glutamine and penicillin/streptomycin (10 U/ml) (Euroclone) at 37°C under a 5% CO2 atmosphere. FH535 (Sigma) and Cyclohexymide (Sigma) were dissolved in DMSO and diluted in colture medium at a concentration of 20 μM. and 25 μM respectively. In both cases, control cells received the same amount of DMSO.

### Plasmids

ZFP36 coding sequences was previously cloned into pCDNA3.1 vector and pRRL lentiviral vector. The ARE-containing regions of the 3′UTRs of MACC1, SOX9 and ZEB1 were amplified by RT-PCR on total RNA extracted from HCT116 cells using specific primers: (MACC1 DP: AGTTCCTCAGGCTTTTTGTCT with MACC1 RP: AGAACTTCACTCTCACTAAACC; SOX9 DP: CCTGTGCCTCTCAGAACACC with SOX9 RP: GCTGGGAGGGAAACAAGTGA; ZEB1 DP: TGGCTCATAACTGTTTCCAAATGT with ZEB1 RP: AAGGCAATAGAAAAAGAAGGCATAA). The amplified fragments were then inserted into the pCR2.1-TOPO T/A cloning vector and fully sequenced. These fragments were then excised with KpnI and XhoI and cloned into pGL3-Promoter Vector (Promega) that had previously been modified in order to transfer the multiple cloning region downstream the luciferase reporter gene. Human Wnt3a coding sequence was amplified by RT-PCR on total RNA extracted from HCT116 cells using specific primers (DP: CGATGGCCCCACTCGGATAC and RP: GCGGCCGGTGCCTACT). The amplified fragments were then inserted into the pCR2.1-TOPO T/A cloning vector and fully sequenced. Wnt3a coding sequences was then excised with XhoI and KpnI and cloned into pCDNA3.1 vector digested with the same enzymes.

### Western blot analysis

Total protein extracts were obtained by resuspending and vortexing cell pellets in small volumes of RIPA buffer containing 50 mM TRIS-HCl (pH 7.4), 150 mM NaCl, 1% NP-40, 1 mM sodium deoxycholate, 1 mM sodium orthovanadate and with added 1 mM EDTA and Complete Protease Inhibitor Cocktail (Roche Applied Science) and quantified. Equal amounts of proteins were loaded onto a SDS-Polyacrilamide gels and transferred to a nitrocellulose sheet. The membranes were then blocked with 5% non-fat milk in TBST 0.1% and immunoblotted overnight at 4°C with different primary antibodies listed below together with their working dilution: N-cadherin (910920 BD Transduction Laboratories); Vimentin (Dako MO725); E-Cadherin (610181 BD); ZO-1 (Zymed 61-7300); MACC1 (Bioss bs-4293R); SOX9 (Bioss bs-4177R); ZEB1 (Sigma HPA027524); ZFP36 (S. Cruz Biotechnology sc-14030). Blots were then incubated either with anti-Rabbit or anti-Mouse (Santa Cruz Biotechnology, sc-2054 and sc-2005 respectively) IgG-HRP antibodies and detected using BM Chemiluinescence Blotting Substrate (Roche Applied Science).

### RNA extraction and qRT-PCR

Total RNA was extracted with PureLink RNA mini Kit (Invitrogen), quantified with Nanodrop 2000 and retro-transcribed with High Capacity cDNA KIT. The obtained cDNA was qPCR quantified using TaqMan Gene Expression Master Mix (Applied Biosystems) with specific primer pairs using the ABI PRISM 7900 HT Detection System (Applied Biosystems). TaqMan Gene Expression assays for GAPDH, ZFP36, TCF7L2 and Axin2 transcripts were purchased from Invitrogen.

### Anchorage-independent growth assays

Anchorage-independent growth assays were performed in triplicate in 35 mm well plates. 72 hours after infection, cells were trypsinised in order to achieve a single-cell suspension and diluted at the same concentrations. Equal amounts of cells were then resuspended in complete DMEM supplemented with 0.3% of Bacto Agar (Sigma) and seeded on the top of a thick layer of DMEM supplemented with 0.6% Bacto Agar. For SW620 and HCT116, 5000 cells were seeded and 10000 for SW480. After 2–3 weeks, colonies were quantified by taking photographs of 10 random fields. For crystal violet staining dishes were fixed with methanol, stained for 3 hours with 0.01% crystal violet in PBS 40% methanol and de-stained with PBS overnight.

### Lentivirus production and infection

Lentiviral particles production was performed as previously described [31]. Briefly, pRRL vectors were co-transfected with packaging plasmids in HEK293T and the resulting supernatant was concentrated through ultracentrifugation at 19000 rpm for 2.5 hours. Target cell lines was infected at MOI 3 overnight with Polybrene. Unless otherwise stated, infected cells were subject to different experimental procedures after 72 hours from infection and with a maximum of one week from infection.

### Anoikis assay

Infected cells were seeded in a 24-wells plate at a density of 200000 cells/well in 0.5 ml of DMEM supplemented with 2 U/ml of Dispase (BD Biosciences) which prevents cell adhesion. Subsequently cells were grown for 72 hours and cellular viability was assessed every day through Trypan blue exclusion assay. Number of cells expansion were normalized on the number of cells seeded at 0 hours.

### Wound healing (scratch) assay

Infected cells were grown in 12-wells plates until complete confluence and then scratched with a p200 pipette tips. Photographs were taken at 0 hours and every day, until gap closure.

### Luciferase assay

For luciferase assays, HEK293T cells were plated at a density of 50000 cells/well the day before transfection in 24-wells plates. In a typical assay, each well received 200 ng of pGL3-based reporter constructs (described in Plasmids chapter), 200 ng of CMV- b-galactosidase plasmid (Clontech Laboratories) and 10 ng of pcDNA3.1 o pCDNA3.1-ZFP36. Transfections were performed using Lipfectamine 2000 (Invitrogen). After 48 hours, cells were harvested and cell lysates were assayed for luciferase and b-galactosidase activity. Luciferase values was normalized on protein concentration and b-galactosidase activity. Each transfection was done in duplicate, and the plotted results represent the average of four different experiments, error bars represent SEM (*p < 0.05; **p < 0.01).

### Immunofluorescence

Briefly, infected cells were grown for 48–72 hours on uncoated coverslips then fixed with 4% paraformaldehyde for 10 minutes and incubated with blocking solution containing 0.1% Saponin (Sigma) and 2% BSA (Sigma) in PBS for 30 minutes. Primary antibody was diluted 1:50 in blocking solution and incubated overnight in humidified chamber at 4°C. Subsequently slides were incubated with fluorescently labelled secondary antibody Alexa Fluor (Invitrogen) and nuclei were counterstained by DAPI (Sigma-Aldrich). Finally, slides were analysed using a Carl Zeiss Axioskop 40 fluorescent microscope.

### Wnt3a conditioned medium production

pCDNA-WNT3a vector (described in Plasmids section) was linearized with SspI and transfected into HCT116 cells. In order to obtain stably transfected cell lines HCT116 was selected with 0.9 μg/ml G418. After 2 weeks of selection, cells were split at 1:8 ratio in absence of G418 and supernatant was collected twice, after 72 h and after further 48 h. The same procedure was performed with pCDNA3.1 empty vector. The so obtained medias were filtered 0,45 μm and kept at –80°C.

### Epithelial barrier functions experiments

The assessment of epithelial barrier functions was performed with Electric Cell-substrate Impedance Sensing (ECIS) Zq instrument (Applied Biophysics). This instrument applies a small alternating current while recording the impedance, and thus the resistance, of the cellular layer. In this case, infected cells were seeded at 10^5^cells/cm^2^ onto uncoated gold microelectrodes in ECIS cultureware (8W10E+) and impedance was recorded immediately after seeding and for at least 48 hours. The results display the resistance recoded at 4000 Hz and normalized on the values recorded at 0 hours and represents the mean of three independent experiments +/−SEM.

### Transwell assays

Infected cells were serum starved for 6 hours and then resuspended in DMEM 0.5% FBS, 0.1% BSA. 60000 SW480 were then seeded in the upper chamber of transwell inserts (8 μm pore and 6.5 mm diameter from Corning) and DMEM 10% FBS was added in the lower chamber in order to provide a chemoattractant stimulus. After 16 hours migrated cells were removed with a cotton swab and the inserts were fixed with cold methanol for 5 minutes and stained with DAPI for 10 minutes. Permeable membranes were then excised with a blade and mounted on a glass slide with mounting medium. Quantification of migrated cells were performed by counting the migrated nuclei on 10 random fields and normalized over pRRL values. Results are the mean of two independent experiments +/−SEM.

### Impedance based scratch assays

Infected cells were seeded on ECIS cultureware (8W1E array) and allowed to reach confluence. Then a wound in the cellular monolayer was generated in by applying a 3 mA, voltage pulse for 20 s, and recovery of the layer was monitored by measuring Resistance at 4000 Hz.

### Statistical analysis

All experiments were repeated at least three times, unless otherwise stated, and the results presented in terms of mean ± S.E.M. values. Pairwise comparisons were carried out using the Student's *t*-test procedure. Results of statistical analysis were considered significant at *p* values < 0.05 (*= < 0.05, **= < 0.001, ***= < 0.0001).

## SUPPLEMENTARY MATERIALS FIGURES AND TABLES


