# Supplementary material for: Loss of zfp36 expression in colorectal cancer correlates to wnt/ β-catenin activity and enhances epithelial-to-mesenchymal transition through upregulation of zeb1, sox9 and macc1
Source: Oncotarget. 2016 Jul 24;7(37):59144–57. doi: 10.18632/oncotarget.10828 (PMC5312301; doi:10.18632/oncotarget.10828)
Supplement: Supplementary file 1 [file oncotarget-07-59144-s001.pdf]

Loss of zfp36 expression in colorectal cancer correlates to wnt/ ß-catenin activity and enhances epithelial-to-mesenchymal transition through upregulation of zeb1, sox9 and macc1

Supplementary Materials

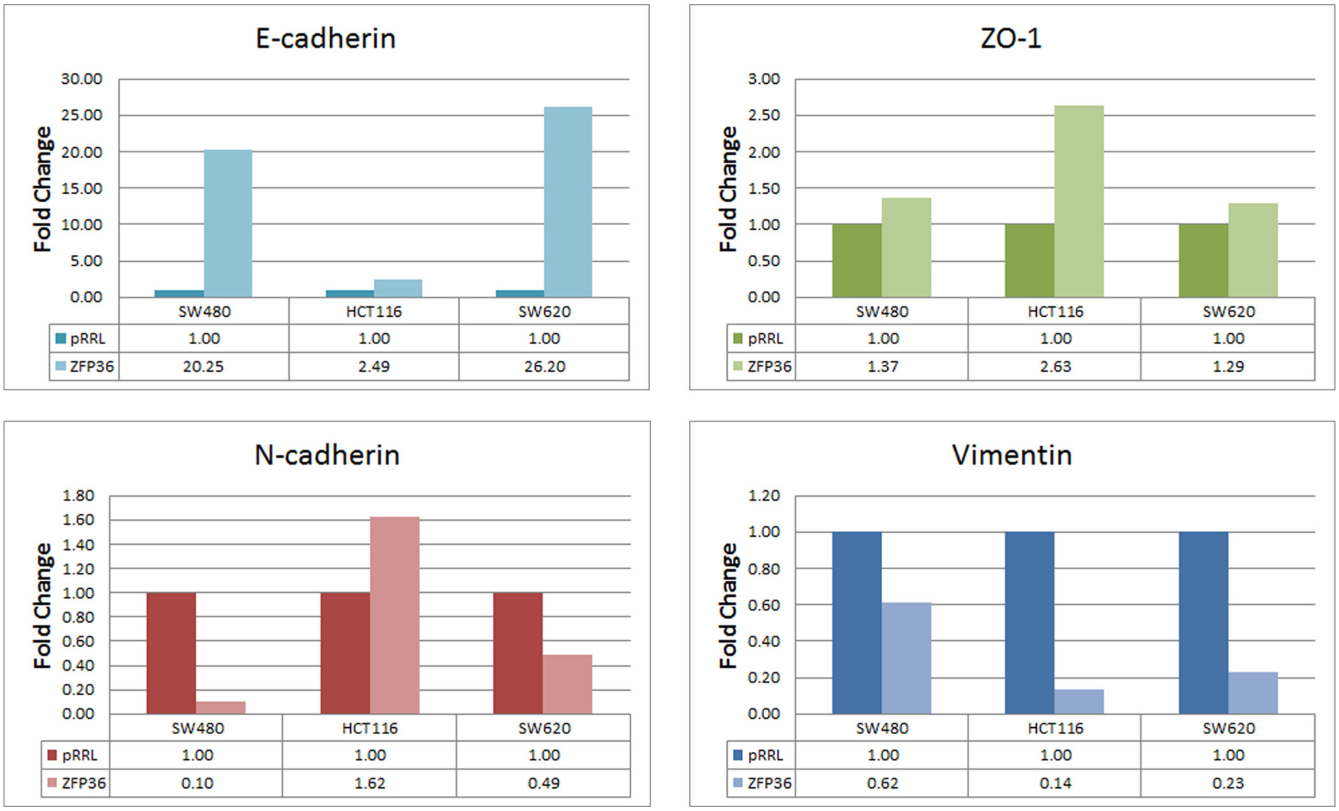

Supplementary Figure S1: Densitometric analysis of E-cadherin, ZO-1, N-Cadherin and Vimentin protein levels in SW480, HCT116 and SW620 infected with empty vector (pRRL) or ZFP36-overepressing vector (ZFP36). Quantification of immuno- reactive bands intensity was carried out with ImageJ software, adjusted on Actin signals and normalized on pRRL values.

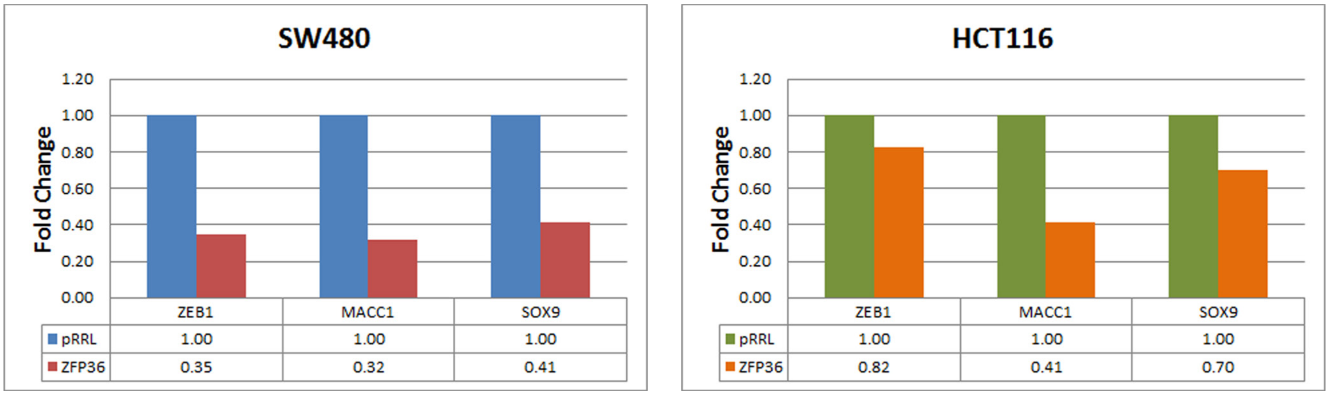

Supplementary Figure S2: Densitometric analysis of ZEB1, MACC1 and SOX9 protein levels in SW480 and HCT116 infected with empty vector (pRRL) or ZFP36- overepressing vector (ZFP36). Quantification of immuno-reactive bands intensity was carried out with ImageJ software, adjusted on Actin signals and normalized on pRRL values.

Supplementary Table S1: Descriptive features of gene expression

	Mean			Median			LogFC		
**Genes**	**Normal**	**CRC**	**Mts**	**Normal**	**CRC**	**Mts**	**CRC *vs* Normal**	**Mts *vs* Normal**	**Mts *vs* CRC**
**ZFP36**	8.774	8.304	8.151	8.722	8.311	8.190	−0.470	−0.623	−0.153
**MACC1**	7.055	8.423	8.495	6.678	8.650	8.622	1.368	1.440	0.072
**SOX9**	8.024	8.645	8.707	7.970	8.757	8.755	0.621	0.683	0.062
**ZEB1**	7.569	6.888	6.576	7.413	6.965	6.488	−0.681	−0.993	−0.313

Normal = normal mucosa, CRC = primary colorectal tumor, Mts = liver metastases, Mean = average mean, LocFC = logarithmic fold change.

Supplementary Table S2: Results of differential expression tests

	Gene	Log FC	Aver.expr	*p*-value	Adj.pP.Val.BH
**CRC *vs* Normal**	ZPF36	–0.470	8.388	1.5E–003	7.66E–003
	MACC1	1.368	8.054	1.69E–006	2.17E–005
	SOX9	0.621	0.621	6.10E–008	1.20E–006
	ZEB1	−0.681	0.979	3.45E–006	4.06E–005
	ZPF36	−0.623	8.388	5.69E–005	4.24E–004
**Mts *vs* Normal**	MACC1	1.440	8.054	9.42E–007	1.22E–005
	SOX9	0.683	8.487	8.88E–009	2.02E–007
	ZEB1	−0.993	6.979	4.15E–010	1.34E–008

LogFC = logarithmic Fold Change, Aver.expr = average expression, Adj.pP.Val.BH = adjusted *p*-value with and Benjamini Hochberg (BH) method, Normal = normal mucosa, CRC = primary tumor, Mts = liver metastases.
